# The Sea Route Planning for Survey Vessel Intelligently Navigating to the Survey Lines

**DOI:** 10.3390/s22020482

**Published:** 2022-01-09

**Authors:** Jiachen Yang, Tianlei Ni, Lin Liu, Jiabao Wen, Jingyi He, Zhengjian Li

**Affiliations:** 1 School of Electrical and Information Engineering, Tianjin University, Tianjin 300072, China; yangjiachen@tju.edu.cn (J.Y.); liulinll@tju.edu.cn (L.L.); Wen_Jiabao@tju.edu.cn (J.W.); lizj@tju.edu.cn (Z.L.); 2 Tianjin International Engineering Institute, Tianjin University, Tianjin 300072, China; nitianlei@tju.edu.cn

**Keywords:** shipborne navigation, survey vessel, survey line, sea route planning, intelligent navigation system

## Abstract

Marine surveying is an important part of marine environment monitoring systems. In order to improve the accuracy of marine surveying and reduce investment in artificial stations, it is necessary to use high-precision GNSS for shipborne navigation measurements. The basic measurement is based on the survey lines that are already planned by surveyors. In response to the needs of survey vessels sailing to the survey line, a method framework for the shortest route planning is proposed. Then an intelligent navigation system for survey vessels is established, which can be applied to online navigation of survey vessels. The essence of the framework is that the vessel can travel along the shortest route to the designated survey line under the limitation of its own minimum turning radius. Comparison and analysis of experiments show that the framework achieves better optimization. The experimental results show that our proposed method can enable the vessel to sail along a shorter path and reach the starting point of the survey line at the specified angle.

## 1. Introduction

As a way to obtain hydrological environment elements, marine surveying is an important part of marine environment monitoring. In the past decade, with the improvement and development of computer technology and information acquisition methods, profound changes have taken place in marine surveying disciplines. Ocean surveying is breaking through the traditional limitation of time and space, entering into the new modern marine measurement stage [1,2]. In the age of digital measurement, 3S (GNSS, GIS [3,4], RS [5,6]) technology is representative. The new technology not only provides high-precision positioning and depth information [7,8,9], but also expands the technical means of obtaining information on oceanographic surveys. For example, in order to improve the accuracy of ocean measurement and reduce the input of artificial stations, researchers around the world are committed to the use of high-precision GNSS [10] for shipborne navigation [11,12] measurement in recent years.

The shipborne navigation measurement mode involves laying out many planned surveys at fixed intervals in the survey area and then surveying along each survey line. It is an important feature of the ocean survey [13,14,15] mode. In this mode of measurement, survey lines’ layout is the core of the technical design of the shipborne navigation measurement system, and it plays a decisive role in ensuring the accuracy and measurement efficiency of the survey results in the entire survey area. On the other hand, it is also particularly important how to lay out a reasonable and the shortest sea route for survey vessels [16] navigating to the survey lines.

According to the physical quantity type, geological structure [17], precision requirements, and mapping data [18], the survey line layout is constantly changing. Therefore, the survey vessel needs to constantly modify the route to steer to the survey line. The route should ensure that the trace of the sea survey vessel is in line with the actual environment, on the condition that the turning radius of the vessel is not less than the minimum turning radius.

Single-beam and multibeam sounding systems [19,20] play an important role in maritime regional surveys, offshore engineering surveys, and waterway surveys. The single beam’s measurement accuracy is not high enough, while the multibeam sounding system not only provides visualization for quantitative monitoring of the waterway area, but also ensures the extensive quantification of monitoring data. At the same time, the multibeam can synthetically measure the complex and diverse underwater terrain data. Therefore, the accuracy of computation and the reliability of a comprehensive analysis of cyclical changes are greatly improved.

**Figure 1:**
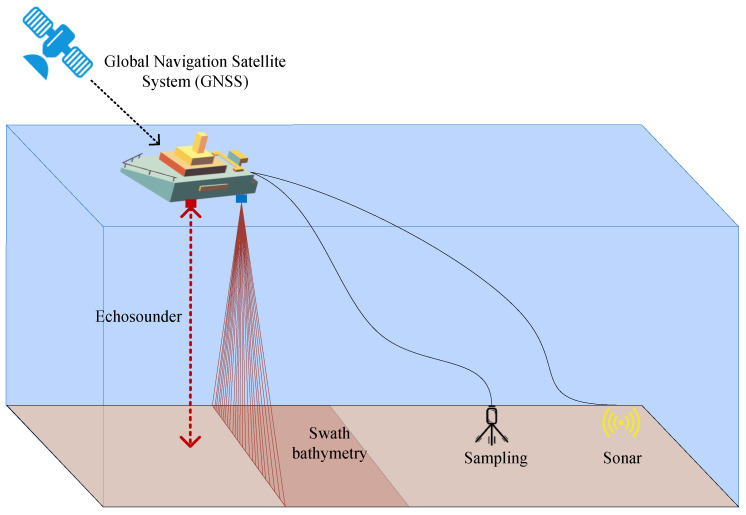
Multibeam echosounder schematic.

International hydrographic standards clearly stipulate that based on the multibeam sounding system [21], we must first ensure full-coverage measurements. On this basis, the greater the sampling point density is, the more perfect the submarine topography will be displayed. The submarine topography delineation depends on the number of sampling points, and the number of sampling points is mainly controlled by the adjustment of survey line spacing. The quality of measurement results cannot be guaranteed by improper spacing selection. Therefore, the survey layout [22] is extremely important and even decisive in the multibeam measurement technology design of the sea. The principle of the multibeam sounder is shown in Figure 1.

The survey layout includes two aspects: survey direction and survey line spacing. Line layout and multibeam measurement are closely related. Therefore, the positions of vessels and lines are random, and the directions of the lines are changeable. After the survey lines are set, the survey vessels will enter the specific area to measure the needed parameters using the sensor, such as the acoustic Doppler current profiler (ADCP) [23] and conductivity, temperature, and depth (CTD) [24,25].

The shortest path problem [26] has always been a research hotspot in the fields of operations research, computer science, geographic information science, and transportation. Many practical problems can be transformed into the calculation of the shortest path in the network through abstraction. For example, the selection of a travel route in the road traffic network and the optimal transmission of information flow between routers in the computer network are related to the calculation of the shortest path.

There are many algorithms to solve the shortest path problem. The traditional algorithms include the Dijkstra algorithm [27] and A* algorithm [28]. The A* algorithm is an effective direct search method for solving the shortest path in a static road network, and it is also an effective algorithm for solving many search problems. In recent years, some new heuristic intelligent algorithms have been proposed, such as the genetic algorithm (GA) [29,30], simulated annealing algorithm (SA) [31,32], tabu search algorithm (TSA) [33], and ant colony algorithm [34,35]. The existing shortest path algorithm mainly studies the shortest path between two designated nodes, the shortest path between all nodes and the shortest path of specifying necessary nodes. When applied in the shipborne navigation measurement, it has two limitations. First, the turning radius of the vessel cannot be less than the minimum turning radius. Second, the arrival direction of the vessel should coincide with the direction of the survey line. Therefore, the traditional shortest path search algorithm is no longer applicable. Navigation personnel often navigate to the survey lines through the GNSS navigation and actual sailing experience, which is not time-saving and economical. Therefore, we propose several better methods.

The goal of this study is to plan reasonable and shortest sea routes for survey vessels that drive into the survey line. To be specific, the most time-saving and distance-saving way that enables survey vessels to quickly enter the starting point of the new planning survey line is expected to be found. The main contributions of this study are as follows:

(1) A methodological framework is proposed for vessels planning the shortest route when the sea survey line changes, which is more time-saving and has greater economic benefits.

(2) We propose different methods to solve the route planning when the vessel is in the far point region or the near point region.

(3) An intelligent navigation system for marine survey vessels is established based on the above framework, which can replace manual surveying and mapping in actual survey tasks, realizing intelligent and unmanned navigation for marine survey ships to adjust survey lines.

The rest of this paper is organized as follows. Section 2 illustrates the proposed methodological framework. Section 3 shows the experimental design and results. The results are discussed in Section 4. Finally, conclusions are drawn in Section 5.

## 2. Methodological Framework

**Figure 2:**
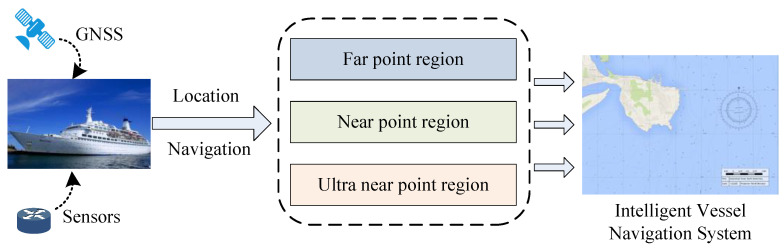
Survey line navigation methodological framework.

This section studies the plan of the survey vessel entering the starting point of the survey line in the survey area. The framework is shown in Figure 2. The coordinate system is re-established with the location of the survey line as a reference, and different routes are designed according to the position and heading angle of the survey ship in the new coordinate system. The path planning algorithm for the survey vessel to the survey line is obtained.

### 2.1. Re-Establish the Coordinate System

**Figure 3:**
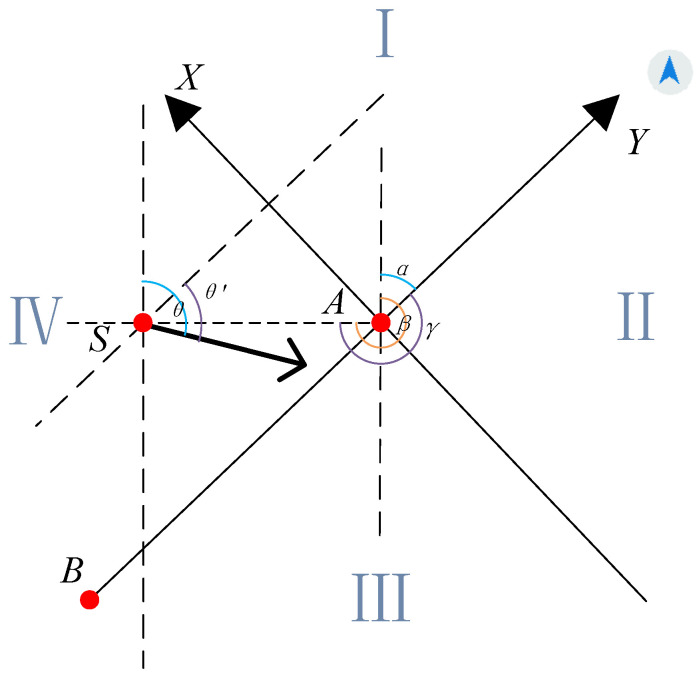
The new coordinate system established according to the direction and position of survey lines.

When the sea survey vessel is near the survey area, it is necessary to establish a mathematical model in order to plan the route of the survey vessel to the survey line. The traveling direction of the vessel is always positioned relative to the true north direction. In order to navigate the vessel toward the survey lines, we re-establish the coordinate system according to the direction and position of the survey lines. The *x*-axis of the established coordinate system coincides with the direction of the survey line, and the *y*-axis is perpendicular to the survey line. The sea area is divided into several different regions, where a different method is provided. The coordinate system, which includes the *x*-axis, *y*-axis, positions of the vessel, and survey line, is shown in Figure 3. Point *A* and point *B* are the starting point and the end point of the survey line, respectively. We have defined some necessary angles to facilitate the calculation of formulas in modeling. α is the angle of $\vec{BA}$ relative to the north. θ is the heading angle of the vessel, whose reference direction is the true north. *δ* is the heading angle of the vessel relative to $\vec{BA}$. β is the angle between the line connecting the vessel’s position and the starting point *A* of the survey line and the true north. γ is the angle between the line connecting the vessel’s position to the starting point *A* of the survey line and the positive direction of the *x*-axis of the new coordinate system. In order to make the distinction easier, we use the expression on the right side of the equation for calculation below. To plan the optimal route for survey vessels, we should know some initial parameters: the position point *S* of the vessel, the starting point *A* and the end point *B* of the survey line, the vessel’s traveling angle θ, and the vessel’s minimum turning radius *R*. Then according to *A*, *B*, and θ, we can calculate α and *δ*.

In the new coordinate system, suppose the coordinate of *A* is $\left(x_{A},y_{A}\right)$, and the coordinate of *B* is $\left(x_{B},y_{B}\right)$. Therefore, the calculation of angle α is shown in Equation (1).

(1) $$\alpha =\{\begin{array}{l} \arctan\frac{x_{A}-x_{B}}{y_{A}-y_{B}},\;\;\;\;\;\;\;\;\;\;\;\;\;\;\;x_{A}>x_{B},y_{A}>y_{B}\\ \pi -\arctan\left|\frac{x_{A}-x_{B}}{y_{A}-y_{B}}\right|,\;\;\;\;\;x_{A}>x_{B},y_{A}<y_{B}\\ \pi +\arctan\left|\frac{x_{A}-x_{B}}{y_{A}-y_{B}}\right|,\;\;\;\;\;x_{A}<x_{B},y_{A}<y_{B}\\ 2\pi +\arctan\left|\frac{x_{A}-x_{B}}{y_{A}-y_{B}}\right|,\;\;\;x_{A}<x_{B},y_{A}>y_{B} \end{array}$$

In the same way, *δ* and γ can be obtained.

(2) δ=(2π+θ−α)mod(2π)

(3) γ=(2π+β−α)mod(2π)

The new coordinate of point *S* can be got according to γ. In the new coordinate system composed of line *AB* as the vertical axis, we can divide four areas by the analogy of the Cartesian coordinate system. Regions I and IV are symmetrical about line *AB* relative to regions II and III, so the paths planned in regions I and IV are also applicable to regions II and III. 

Therefore, in this study we will only discuss situations when the vessel (Point *S*) is in region I and region IV. Then all methods to get routes can be applied in region II and region III. Unless otherwise specified, all the following routes we propose are under the new coordinate system. Since survey vessels will be close to the area needed to survey when working, we suppose that the area that includes survey vessels and survey lines is flat, and the curvature of the earth is neglected.

### 2.2. Redistribution of the Navigable Area

**Figure 4:**
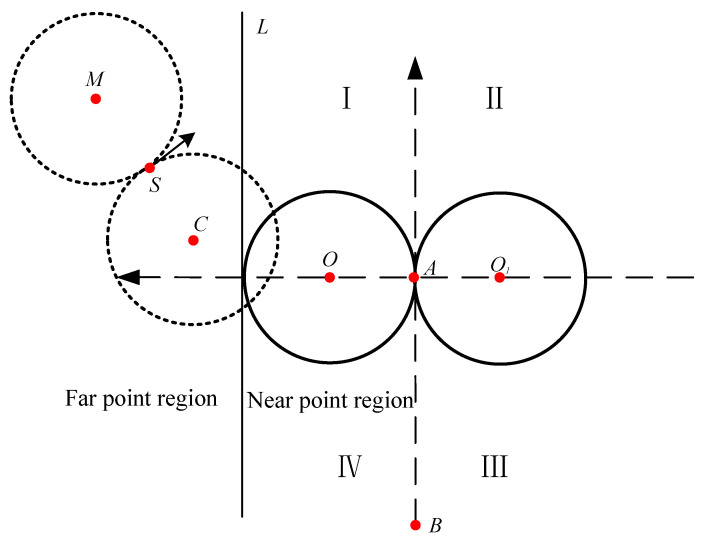
Redistribution of the navigable area.

The radius of this circle is the minimum turning radius *R*. Taking *R* as a reference, some definitions in Figure 4 can be obtained.

Circle C: The vessel rotates clockwise, while the rotating radius is *R*. That is, the starboard side of the ship turns to circle *C*. Its coordinate is $\left(x_{C},y_{C}\right)$.

Circle M: The vessel rotates anticlockwise, while the rotating radius is *R*. That is, the port side of the ship turns to circle *M*. Its coordinate is $\left(x_{M},y_{M}\right)$.

Circle O: The circle on the left of survey line *AB* in the new coordinate system and the radius of it is *R*. Its coordinate is $\left(x_{O},y_{O}\right)$.

Circle $O_{1}$: The circle on the right of survey line *AB* in the new coordinate system and the radius of it is *R*. Its coordinate is $\left(x_{O_{1}},y_{O_{1}}\right)$.

Line L: Line L and line *AB* are parallel. The distance between line L and survey line *AB* is 2*R*. Line L is used to distinguish near and far points due to the necessity of reallocating navigable areas.

The area is redivided according to the location and direction of survey lines. Next, we will give different routes for different areas. After obtaining the initial parameters, we could calculate the distance from point *S* to the line formed by point *A* and point *B* to get the position and direction of the survey vessel in a new coordinate system. Then the corresponding navigation route can be given. 

### 2.3. Far Point Region Analysis

**Figure 5:**
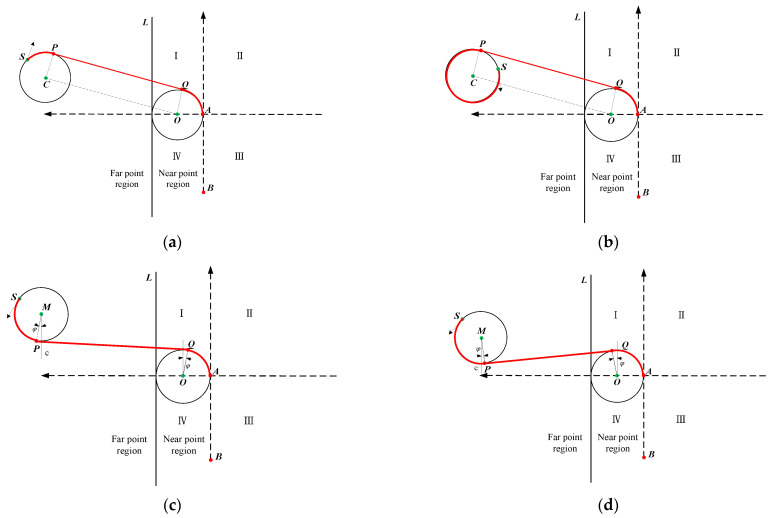
There are four routes in far point region I: (**a**) turning to starboard (route 1), (**b**) turning to starboard when the vessel is to the left of point *P* (route 1′), (**c**) turning to port when $y_{M}\geq y_{O}+2R$ (route 2), and (**d**) turning to port when $y_{M}<y_{O}+2R$ (route 2′).

When the vessel is in the far point region, the vertical distance from the measuring line is generally greater than the turning diameter of the vessel at this time, so the vessel can always enter the measuring line smoothly. There are two options of starboard steering and port steering, which are shown in Figure 5. We named the two routes turning to starboard in region I as route 1 and route 1′ and the two routes turning to port in region I as route 2 and route 2′. All routes planned are marked in red and the arrow at point *S* indicates the heading of the vessel in Section 2.

In route 1, PQ||CO, CP⊥PQ, OQ⊥PQ, and PQ are tangent to circle O and circle C. The essence of route 1 is to find a common tangent of circle O and circle C. 

The total distance of route 1 is

(4) $$d_{route1}=(\pi -\delta )*R+d_{CO}$$

where $d_{CO}$ is the distance between point *C* and point *O*, which is equal to the distance between point *P* and point *Q*. (π−δ) is equal to the sum of central angles corresponding to two arcs (arc *SP* and arc *AQ*).

Make a straight line, which is parallel to the *x*-axis through point *C*. This straight line divides circle *C* into upper and lower parts. As shown in Figure 5b, when the vessel is in the upper half and to the left of point *P*, $d_{route1}$ can be calculated by Equation (4). When the vessel is in another position of circle *C*, we need to add 2π to the angle value when calculating the length of route 1.

Actually, the routes planned when $x_{C}<-R$ are equivalent to that when $x_{C}\geq -R$. Therefore, in this paper we will only discuss situations when $x_{C}<-R$. Similarly, only one case is discussed in the near point region and the ultra near point region.

In route 2, MP⊥PQ, OQ⊥PQ, and PQ are tangent to circle M and circle O. Similarly, the essence of route 2 is to find a common tangent of circle M and circle O. Different from route 1, route 2 is to make the vessel rotate anticlockwise to the common tangent of circle M and circle O. To get the total distance of route 2, we defined a middle angle named φ. The relative position of circle M and circle O in the new coordinate system distinguishes four situations; two of them are shown in Figure 5c,d.

As shown in Figure 5c, when $y_{M}\geq y_{O}+2R$ and $x_{M}\geq -R$,

(5) $$\varphi =\arccos\frac{2R}{d_{MO}}-\arctan\left|\frac{x_{M}-x_{O}}{y_{M}-y_{O}}\right|$$

where $\arccos\frac{2R}{d_{MO}}$ is the angle between the line connecting point *M* to point *O* and line *MP*, and $\arctan\left|\frac{x_{M}-x_{O}}{y_{M}-y_{O}}\right|$ is the angle between the line connecting point *M* to point *O* and line *c*.

Angle φ is marked in Figure 5c. At this time, the coordinate of point *P* is $\left(x_{M}+R*\sin\varphi ,\;y_{M}-R*\cos\varphi \right)$, and the coordinate of point *Q* is $\left(x_{O}-R*\sin\varphi ,\;y_{O}+R*\cos\varphi \right)$. Therefore, the total distance of route 2 in different situations can be obtained.

(6) $$d_{route2}=\{\begin{array}{cc} (2\pi +\delta -2\varphi )*R+d_{PQ}, & 0\leq \delta <\frac{\pi }{2}+\varphi \\ (\delta -2\varphi )*R+d_{PQ}, & \frac{\pi }{2}+\varphi \leq \delta <2\pi \end{array}$$

The reason for using $\frac{\pi }{2}+\varphi$ as the critical value of the function segmentation is that when δ is less than $\frac{\pi }{2}+\varphi$, the vessel is on the right side of point *P* on circle *M*. The vessel needs to make a full rotation along the port side of circle *M* and return to point *P* before continuing to point *A*. Therefore, it is necessary to add 2π when calculating the arc length. $d_{PQ}$ is the distance between point *P* and point *Q*.

Similarly, as shown in Figure 5d, when $y_{M}<y_{O}+2R$ and $x_{M}\geq -R$,

(7) $$\varphi =\arctan\left|\frac{x_{M}-x_{O}}{y_{M}-y_{O}}\right|-\arccos\frac{2R}{d_{MO}}$$

In this case, the distance of route 2′ can be calculated on the same principle as that of route 2.

(8) $$d_{route2^{\prime }}=\{\begin{array}{cc} (2\pi +\delta +2\varphi )*R+d_{PQ}, & 0\leq \delta <\frac{\pi }{2}+\varphi \\ \;(\delta +2\varphi )*R+d_{PQ}, & \frac{\pi }{2}+\varphi \leq \delta <2\pi \end{array}$$

**Figure 6:**
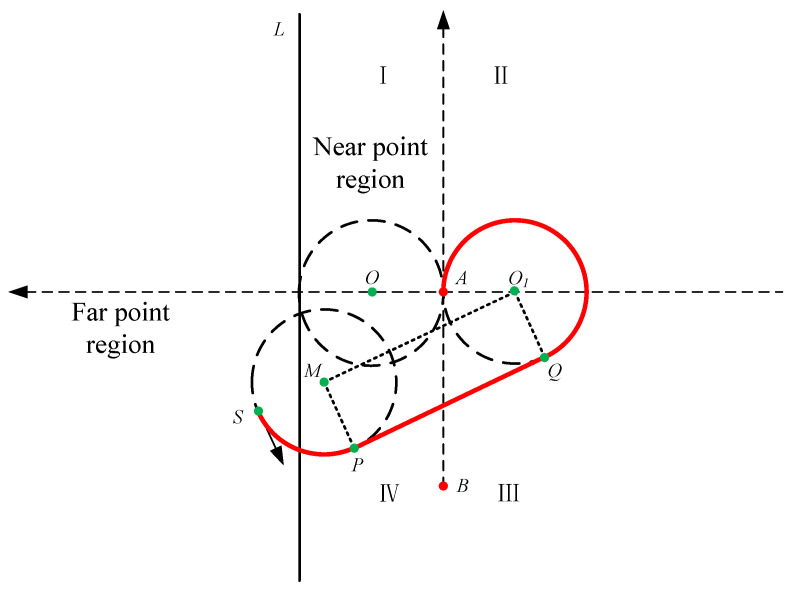
The situation where the far point region is in region IV (route 1″).

When the vessel drives into the far point region in region I, basic parameters are obtained. Then we compare the distances sailing along two routes to get the shortest path. Further, when the far point region is in region IV, as shown in Figure 6, a route named route 1″ should be compared with other two routes to get the shortest path. The relative position of M and $O_{1}$ distinguishes different situations. The total distance of route 1″ is

(9) $$d_{route1^{\prime \prime }}=(\delta -\pi )*R+d_{MO_{1}}$$

where $d_{MO_{1}}$ is the distance between point *M* and point $O_{1}$, which is equal to $d_{PQ}$. (δ−π)∗R is the sum of the arc length on circle M and circle $O_{1}$. According to the relative position of circle M and circle $O_{1}$, we need to add 2π or 4π when calculating the radian value to get the arc length.

### 2.4. Near Point Region Analysis

Route 2 can only be used when $d_{MO}\geq 2R$ and $x_{M}\geq -R$, so we will still split the near point region into two regions, which are general region and ultra near point region.

The near point area is suitable for the scenario where the vessel switches to another survey line for measurement after measuring one survey line. Therefore, the planning of the near point area oceanographic survey vessel’s approach to the survey line path is of great significance in the shipborne navigational survey mode.

In the general near point region, $d_{MO}\geq 2R$ and $x_{M}\geq -R$ exist or $d_{CO_{1}}\geq 2R$ and $x_{c}\leq R$ exist. When $d_{MO}\geq 2R$, $x_{M}\geq -R$, and point *S* is in region I, route 1 and route 2 can be used for the survey vessels to sail to the starting point *A* of survey lines. $d_{route1}$ and $d_{route2}$ are compared to get the shortest path. When $d_{MO}\geq 2R$, $x_{M}\geq -R$, and point *S* is in region IV, $d_{route1}$, $d_{route2}$, $d_{route1^{\prime }}$, and $d_{route2^{\prime }}$ are compared to get the shortest path.

**Figure 7:**
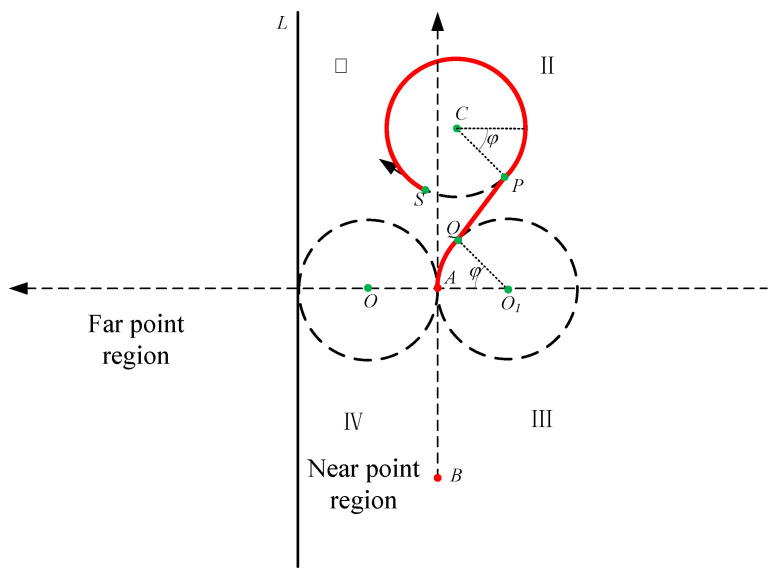
Route 3 in the near point region.

However, the position of some points cannot meet the condition of $d_{MO}\geq 2R$ and $x_{M}\geq -R$. On the contrary, the conditions $d_{CO_{1}}\geq 2R$ and $x_{C}\leq R$ and are met. Therefore, we plan a new route named route 3 in Figure 7. In route 3, CP⊥PQ, $O_{1}Q⊥PQ$, and PQ are tangent to circle C and circle $O_{1}$. The essence of route 3 is to find a common tangent of circle $O_{1}$ and circle C. It is obvious that route 3 is the shortest route in this situation. We define φ in Figure 7.

(10) $$\varphi =\arctan\left|\frac{y_{C}-y_{O_{1}}}{x_{C}-x_{O_{1}}}\right|-\arccos\frac{2R}{d_{CO_{1}}}$$

Therefore, the distance of route 3 is

(11) $$d_{route3}=\{\begin{array}{l} (\pi -\delta +2\varphi )*R+d_{PQ},\;\;\;0\leq \delta <\pi +\varphi \\ (3\pi -\delta +2\varphi )*R+d_{PQ},\;\pi +\varphi \leq \delta <2\pi \end{array}$$

where when π+φ≤δ<2π, the vessel needs to make one more round along circle *C* and return to point *P* again. Therefore, there is 2π more when calculating the arc length.

### 2.5. Ultra near Point Region Analysis

**Figure 8:**
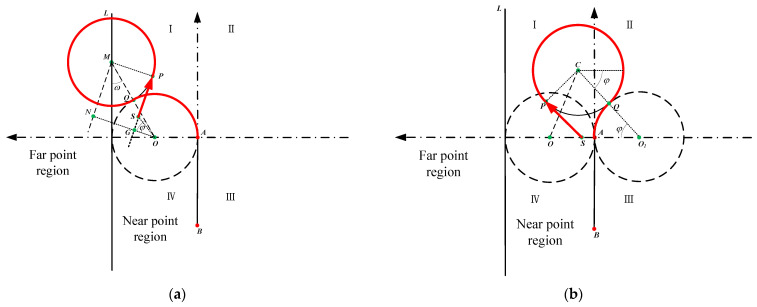
Routes of the ultra near point region: (**a**) route 4; (**b**) route 5.

In ultra near point region I, the condition $d_{MO}\geq 2R$ or $x_{M}\leq -R$ is met. Therefore, route 2 is no longer applicable. In some situations, especially when 0≤δ<2π, route 1 is not the optimal route. We plan a new route named route 4 for survey vessels to run a straight line until $d_{MO}=2R$. Routes of the ultra near point region are represented in Figure 8.

In route 4, MN||SP, ON||MP, ON⊥MN, and SP are tangent to circle M. $x_{S}>R$, φ=∠MON. To get *φ*, we should know whether line SP is higher or lower than the vertical line passing $\left(x_{O},y_{O}\right)$ when $x_{S}>R$. To distinguish two situations, the angle of $\vec{OS}$ relative to $\vec{AB}$ is defined as ω. All auxiliary angles can be obtained from the coordinates of the points.

(12) $$d_{route4}=\{\begin{array}{l} (\frac{5\pi }{2}-\delta -\omega )*R+d_{SP},\;x_{M}\leq x_{O}\\ (\frac{5\pi }{2}-\delta +\omega )*R+d_{SP},\;x_{M}>x_{O} \end{array}$$

After the calculation in MATLAB software, we found that in ultra near point region I and $0\leq \delta <\frac{\pi }{2}$, $d_{route4}$ is always shorter than $d_{route1}$. In region I and $\frac{\pi }{2}\leq \delta <\pi$, route 1 is the shortest. In region I and π≤δ<2π, it is clear that route 1 is the shortest route when $x_{C}\leq R$. However, when $x_{C}>R$, a new route named route 5 is used to lay out the shortest route for survey vessels. In route 5, *CP* ⊥ *SP*. The core of route 5 is moving the position of the vessel to make circle C tangent to circle $O_{1}$. It is obvious that route 5 is the shortest route in this situation. Route 5 is applicable to the situation where the vessel is in region I, $x_{C}\leq R$, and the heading angle range is (*π*, 2*π*). The total distance of route 5 can be obtained by

(13) $$d_{route5}=\{\begin{array}{c} (\pi -\delta +2\varphi )*R+d_{SP},\;0\leq \delta <\pi +\varphi \\ (3\pi -\delta +2\varphi )*R+d_{SP},\;\pi +\varphi \leq \delta <2\pi \end{array}$$

Similarly, 2π is added when π+φ≤δ<2π because of one more round along circle *C*.

### 2.6. Intelligent Vessel Navigation System

**Figure 9:**
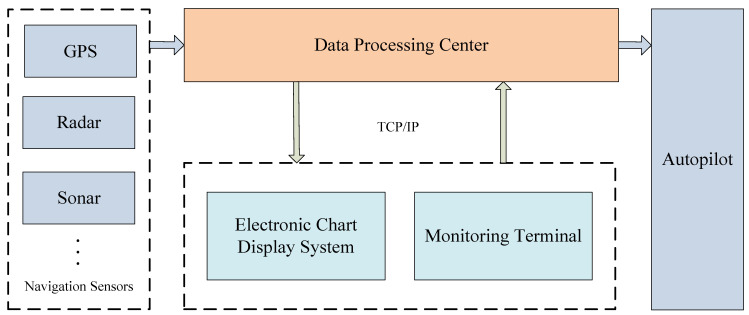
The framework of an intelligent vessel navigation system.

Based on the methods proposed above, an intelligent vessel navigation system is designed in this study. The framework of this system is shown in Figure 9. The system obtains the vessel’s position information from the navigation sensors. Combining the survey line with the position and direction of the vessel, the corresponding route planning algorithm will be selected to help navigators carry out route design and route monitoring so as to get the shortest route. In the past, the crew could only use paper sea charts when sailing at sea. However, paper sea charts highly depend on human surveying and mapping, which greatly increases the work intensity of the crew and is not conducive to the safety of vessel navigation. The intelligent vessel navigation system designed in this study can realize vessel route planning without human intervention, which provides great convenience for the crew to navigate. Besides, the intelligent vessel navigation system can display electronic sea charts, in which the planned route and the current route can guarantee the accuracy of navigation.

## 3. Experimental Results

**Figure 10:**
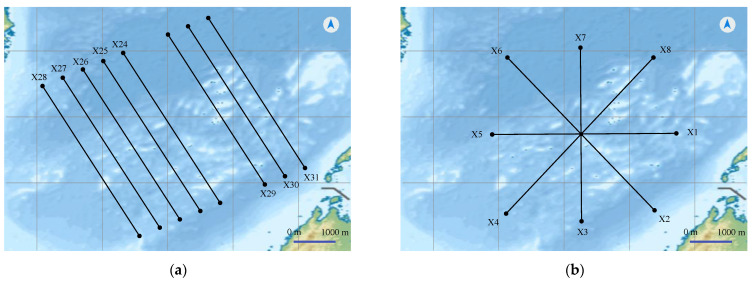
Components of the main line layout: (**a**) parallel survey lines; (**b**) star survey lines.

The experiments in our study are mainly carried in MATLAB software on Ubuntu 20.04.3 LTS, with Intel^®^ Xeon^®^ CPU E5-2620 v4 @ 2.10 GHz × 32 CPU and NVIDIA TITAN Xp GPU. In this study, the routes are added to the above intelligent vessel navigation system, whose background is electronic sea charts. It is more intuitive to display the navigation sea route for survey vessels. The sea survey line consists of the main line and the inspection line. Its layout is essential for effective detection of the sea floor. In the actual measurement work, in order to further detect some specific submarine targets, we still need to add encrypted survey lines based on the main survey lines. Parallel lines and star lines in Figure 10 make up the main line layout. The hydrographic survey specifications stipulate that the angle between the main survey line and the inspection line should be controlled between 45° and 90°. The specifications require that survey lines should be laid out in a flat place. Ideally, we set the main line and the inspection line orthogonally. The main purpose is to use the shortest mileage inspection line to achieve the general inspection of the main line.

**Figure 11:**
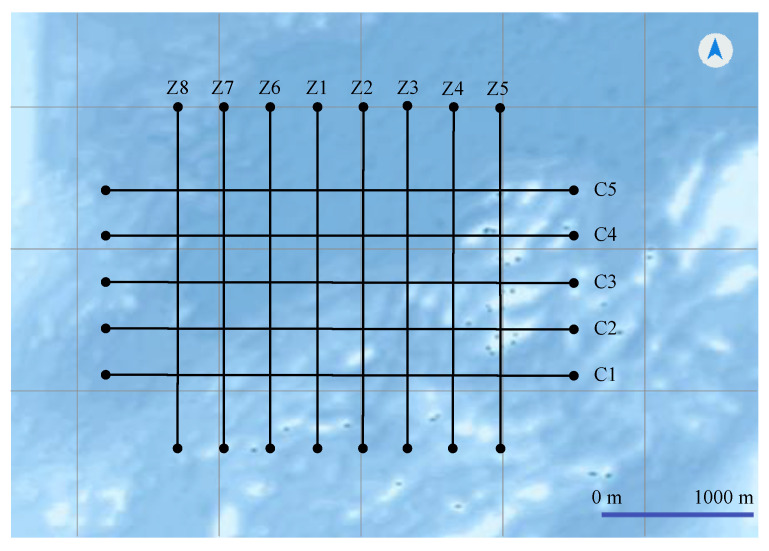
The parallel survey lines in an intelligent vessel navigation system.

**Figure 12:**
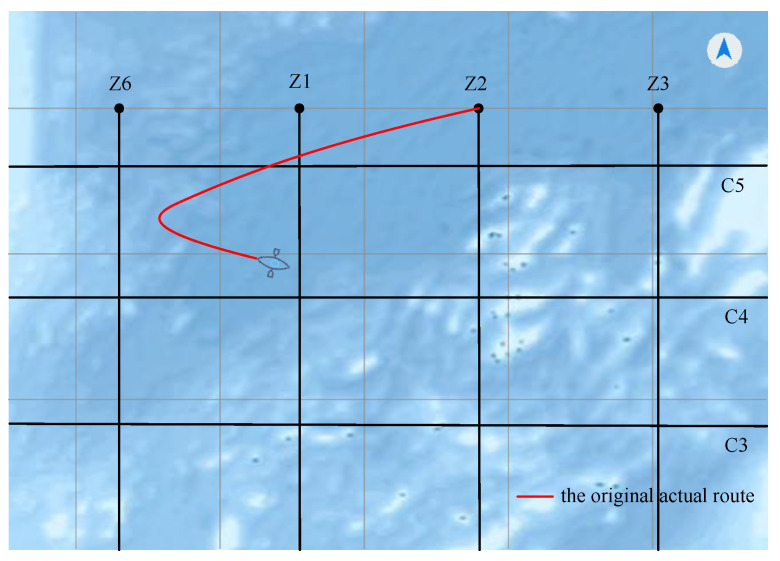
The original system’s navigation trajectory.

First, we lay out eight parallel main survey lines and five inspection lines in the simulated sea navigation system. As shown in Figure 11, the interval between the eight main lines is about 300 m, and the direction of the main lines is 180°. Then we assume that the minimum turning radius of the sea survey vessel is 100 m. Figure 12 is a schematic diagram of the original navigation system with an unreasonable navigation trajectory. The trajectory does not take the actual turning radius of the survey vessels into consideration. When the vessel reaches survey line Z2, it cannot adjust the vessel’s direction to make it drive along Z2. Obviously, the original navigation route cannot be applied to the actual navigation. 

**Figure 13:**
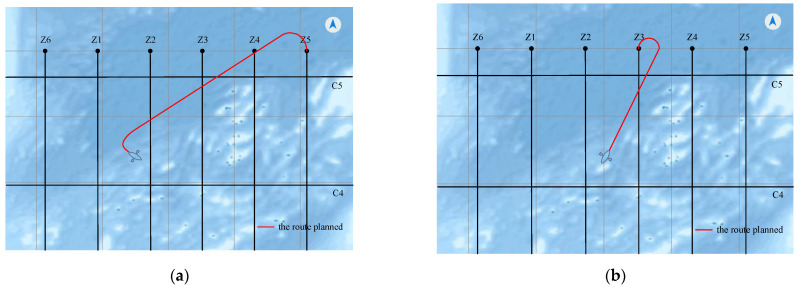
Navigation sea routes in an intelligent vessel navigation system: (**a**) the navigation trajectory used route 1; (**b**) the navigation trajectory used route 1″.

Second, we add the routes planned in this study into the navigation simulated system, and the navigation sea routes of different situations can be seen in Figure 13. The first situation where θ=300° and the survey vessel is in the far point region shows that the navigation line used route 1, which will navigate the vessel drive into survey line Z5. The second situation where θ=30° and the vessel is in the general region of the near point region shows the navigation line route 1″, which will navigate the vessel drive into survey line Z3.

The data are mainly measured based on line AB in the main survey lines. Table 1 indicates some test points. The minimum turning radius is 100 m. The latitude and longitude of the points are expressed in coordinates for calculation. Table 2 shows the total distance of each sea route proposed in this study.

Then we compare the proposed methods with other practical schemes. In the far point region, the survey vessel is able to swivel before reaching the survey line. It can turn along an arc whose radius is greater than R to make the trajectory tangent to the extension of the survey line. Experiments show that the distance traveled using this method is significantly greater than the method we proposed. Similarly, turning by the minimum turning radius while going straight is always the best way to get the shortest route ideally. The position of the survey line and the minimum turning radius in Table 3 are the same with those in Table 1. Table 4 shows the comparison results. Obviously, the methods we proposed are shorter than other methods in the navigation system.

**Figure 14:**
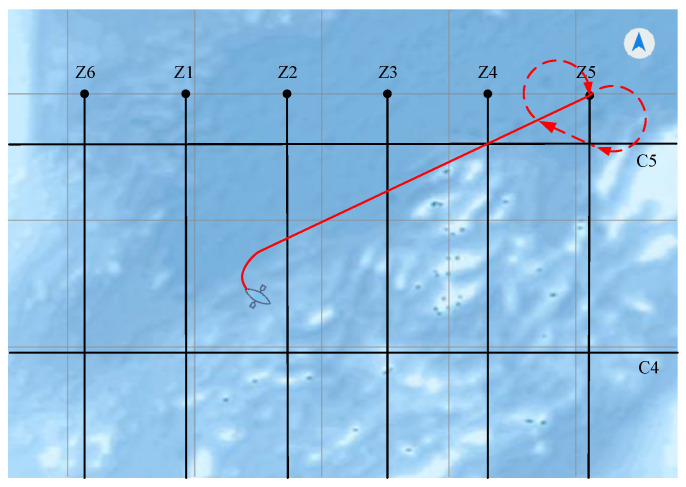
The route planned with the improved ant colony algorithm. The red solid line represents the planned route for the vessel to reach the starting point of the survey line, and the red doted line represents the planned route for the vessel to adjust its heading from the starting point of the survey line.

In order to verify the effectiveness of the proposed method, we compared it with other state-of-the-art methods. Luo et al. [35] proposed an improved ant colony algorithm, which uses a pseudo-random state transition rule to select the path and introduces the optimal solution and the worst solution, dynamic punishment method. The global optimal search ability and convergence speed of the improved algorithm have been greatly improved. We apply the improved ant colony algorithm to the navigation of a marine survey vessel. We select the situation where the vessel is in the far point region. The experimental result is shown in Figure 14. When the vessel reaches the starting point of the survey line for the first time, the length of the passing route marked by the red solid line is 1332.4 m, which is shorter than 1564.2 m planned by the method we proposed. Although the route planned by the traditional path planning algorithm is shorter, it cannot meet the requirements of line survey navigation because of the lack of consideration of the vessel’s navigation angle. When the vessel reaches the starting point of the survey line, its heading does not coincide with the survey line direction. The vessel needs to turn to adjust its heading angle to be consistent with the survey line, as shown by the red dotted line, which is 1016 m. Therefore, the total length of the route planned by the improved ant colony algorithm is 2348.4 m, which is longer than 1564.2 m planned by the method we proposed.

## 4. Discussion

It can be seen from the experimental results in Table 2 that the distance of the vessel’s navigation route is not directly related to whether it is in the far point region or the near point region. The route is not necessarily longer when the vessel is in the far point region, and not necessarily shorter when the vessel is in the near point region. The heading angle of the vessel has an important influence in the navigation of the survey line. Additionally, due to the randomness of the experiment, the route length planned each time may not be exactly the same. In order to verify the accuracy and stability of the method, we selected the super near point region and repeated the experiment 10 times. The experimental results are shown in Table 5. All conditions being the same, the path length is maintained between 635 and 661 m. The error does not exceed 2%, which shows that our method is accurate.

We take the ultra near region as an example and verify the effectiveness of the proposed method for line survey navigation by simulating past actual route cases.

**Figure 15:**
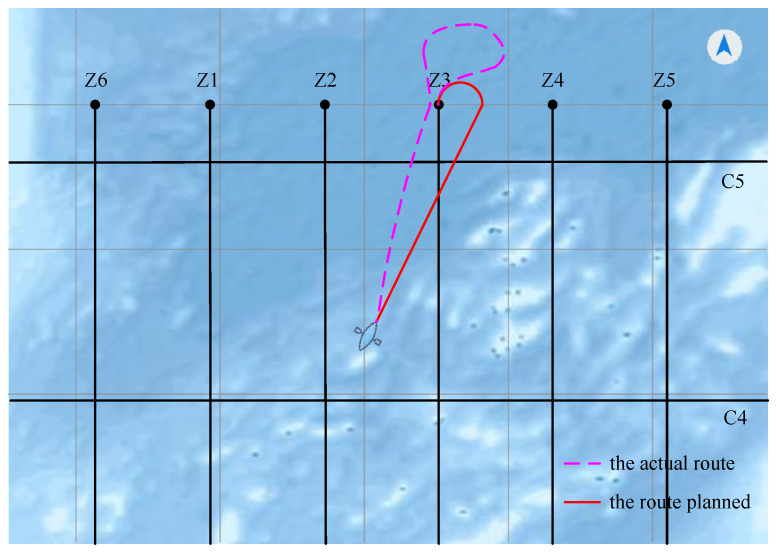
Comparison of the route planned by the proposed method and the actual situation.

On 2 June 2015, the vessel was located between the two survey lines Z2 and Z3, with a heading angle of 30°. Using the conditions at the time as the background, the navigation was simulated, and the difference between the proposed method and the actual situation was compared (Figure 15). The red line represents the path we planned, and the yellow line represents the actual situation. It can be seen from the figure that the strategy of vessel selection is different in the two cases. In the actual situation, the vessel first traveled in the direction of the shortest path towards the starting point of the survey line. Later, when it was about to reach the starting point of the survey line, the vessel had to adjust its heading by making a circle, which greatly increased the path length. In our method, the vessel does not drive directly towards the starting point of the survey line, and the heading angle is taken into account at the beginning, which reduces the path a lot.

Ocean surveying is an important part of the science of surveying and mapping. Its main task is to accurately measure and describe the geometric and physical parameters of the ocean and provide necessary oceanographic data for human activities to help humans better understand the ocean and make rational use of marine resources. In the shipboard navigation measurement mode, survey vessels can sail along the designated survey line to measure the value, distribution, and change of marine hydrological elements, such as temperature, salinity data, and microbial information of a given sea area.

There are two main ways to measure hydrological elements. The first is direct observation. This method uses the sensing elements in the equipment to directly measure hydrological elements, utilizing ships and buoys as carriers. The second method is remote sensing observation. In this way, aircraft, satellites, and so forth are used as carriers. Radars are used to detect and record the electromagnetic radiation information of the ocean and extract the characteristics of ocean hydrological elements. This study proposes several methods of route planning for the ship survey in the first survey method. We abandon the old uneconomical and inaccurate method of measuring ships sailing along the survey line and plan the shortest route according to different situations so that the work is more time-saving and brings greater economic benefits. After the simulation of the intelligent vessel navigation system, it is found that the route planning method proposed in this study has a shorter distance.

On the other hand, route planning is one of the key technologies for ensuring the successful completion of the mission of the survey ship. In actual route planning, there are many indicators to evaluate the quality of route planning, such as the voyage between the starting point and the target point, safety, and quality of information acquisition. In the process of solving the route planning problem, we need to consider not only the constraints of the platform itself but also the constraints of ocean currents, obstacles, and terrain. The framework proposed in this study only considers the shortest voyage issue without considering other constraints. Moreover, it is promising to apply the intelligent algorithm of path planning to ship route planning. At present, the methods used for underwater route planning can be roughly divided into graph search type, random sampling type, analysis type, intelligent type, and combined optimization type. Currently, the algorithms widely used in route planning are intelligent algorithms, including ant colony optimization algorithm and particle swarm optimization algorithm. It is a research direction to apply intelligent algorithms to marine survey vessel route planning.

## 5. Conclusions

In this study, we proposed a methodological framework that is suitable for sea survey ships to change survey lines and designed an intelligent vessel navigation system. When the sea survey vessel enters the area to be measured, the coordinate system is re-established according to the direction and position of the survey line. The region is divided to the far point region and near point region, which includes the general near point region and ultra near point region. Different route planning schemes are adopted according to the position and heading angle of the sea survey vessel. The coordinates of points in the new coordinate system are calculated to draw navigation lines. According to the different position of the vessel, we plan appropriate routes, such as route 1 and route 1′, whose total distance is given. Additionally, the sea chart simulation is used to display intuitively. We conducted experimental simulations on different situations and compared them with the improved ant colony algorithm and actual navigation conditions. It can be found that the proposed method can effectively consider the heading angle of the sea survey vessel when entering the survey line, avoiding a longer route. Moreover, through repeated experiments under the same conditions, it is found that the route length error planned by the method in this study is small, which verifies the stability and accuracy. Finally, we designed an intelligent vessel navigation system that can help the vessel to plan the route for entering the sea survey line more effectively by obtaining the vessel’s position and heading information.

However, factors such as islands, ships, and ocean currents, which are inevitable in actual work, were not considered in navigation planning. In the future, we will add routes to the real marine vessel navigation system and take the actual factors as mentioned before into account to fix the navigation routes.

## Figures and Tables

**Table 1:** Test points that are in different situations.

Number	*S* ^1^	$\theta {\;}^{2}$	*A*	*B*	Region
1	(200, 500)	0°	(700, 400)	(700, 100)	Far point region
2	(200, 500)	180°	(700, 400)	(700, 100)	Far point region
3	(630, 300)	180°	(700, 400)	(700, 100)	Far point region
4	(670, 500)	270°	(700, 400)	(700, 100)	General near point region
5	(650, 420)	60°	(700, 400)	(700, 100)	Ultra near point region
6	(680, 420)	290°	(700, 400)	(700, 100)	Ultra near point region

^1^*S* is the coordinate of the sea survey vessel. ^2^ θ is the driving direction of the vessel relative to the north.

**Table 2:** The real data of different routes for the test points in Table 1.

Number	*R*/m	Routes	Estimated Distance/m
1	100	Route 1	630.39
2	100	Route 2	631.70
3	100	Route 1″	750.38
4	100	Route 3	684.85
5	100	Route 4	743.68
6	100	Route 5	650.67

**Table 3:** Test points chosen for comparison.

Number	*S*	θ	*A*	*B*	*R*/m
1	(100, 500)	0°	(700, 400)	(700, 100)	100
2	(100, 500)	180°	(700, 400)	(700, 100)	100
3	(620, 200)	150°	(700, 400)	(700, 100)	100

**Table 4:** Comparison between the proposed methods and other practical schemes.

Number	Routes	Distance/m	Other Methods	Distance/m	Evaluation on Proposal
1	Route 1	726.47	Turning radius > R	1042.48	shorter
2	Route 2	727.01	Route 1	1236.59	shorter
3	Route 1″	752.66	Route 1	939.40	shorter

**Table 5:** Repeated experiments in the super near point area.

Number	*S*	θ	*A*	*B*	Distance/m
1	(680, 420)	290°	(700, 400)	(700, 100)	645.67
2	(680, 420)	290°	(700, 400)	(700, 100)	638.20
3	(680, 420)	290°	(700, 400)	(700, 100)	652.37
4	(680, 420)	290°	(700, 400)	(700, 100)	651.28
5	(680, 420)	290°.	(700, 400)	(700, 100)	658.95
6	(680, 420)	290°	(700, 400)	(700, 100)	650.14
7	(680, 420)	290°	(700, 400)	(700, 100)	661.44
8	(680, 420)	290°	(700, 400)	(700, 100)	650.78
9	(680, 420)	290°	(700, 400)	(700, 100)	647.19
10	(680, 420)	290°	(700, 400)	(700, 100)	636.56

## Data Availability

Not applicable.
